# The predictive role of the dietary phytochemical index in relation to the clinical and psychological traits of migraine headaches

**DOI:** 10.1038/s41598-024-57536-7

**Published:** 2024-03-22

**Authors:** Shahnaz Amani Tirani, Arghavan Balali, Maryam Kazemi, Gholamreza Askari, Fariborz Khorvash, Arman Arab

**Affiliations:** 1 Student Research Committee, Isfahan University of Medical Sciences, Isfahan, Iran; 2 Department of Community Nutrition, School of Nutrition and Food Science, Nutrition and Food Security Research Center, Isfahan University of Medical Sciences, Isfahan, Iran; 3 Department of Nutrition, Harvard T.H. Chan School of Public Health, Boston, MA USA; 4 Isfahan Neurosciences Research Center, Alzahra Hospital, Isfahan University of Medical Sciences, Isfahan, Iran; 5 Division of Sleep Medicine, Harvard Medical School, Boston, MA USA; 6 Medical Chronobiology Program, Division of Sleep and Circadian Disorders, Department of Medicine and Neurology, Brigham and Women’s Hospital, 221 Longwood Ave., Boston, MA 02115 USA

**Keywords:** Migraine disorders, Headache, Diet, Dietary phytochemical index, Neurology, Nutrition

## Abstract

We investigated the relationship between dietary phytochemical index (DPI) and migraine headaches in Iranian patients, analyzing both clinical and psychological traits. A cross-sectional study was conducted using non-obese adults aged 20–50 years who were diagnosed with migraine. The study used a validated 168-item food frequency questionnaire to assess the usual dietary intake of participants. The DPI was calculated using the following formula: [daily energy derived from phytochemical-rich foods (in kJ)/total daily energy intake (in kJ)] × 100. Clinical outcomes of migraine including frequency, duration, and severity of headaches, as well as migraine-related disability were obtained using relevant questionnaires. Moreover, the mental health profile of patients including depression, anxiety, and stress, as well as serum levels of nitric oxide (NO) were measured. A Poisson regression was used for headache frequency. Linear regression analyzed migraine-related outcomes including duration, severity, migraine-related disability, and serum NO levels. In addition, psychological traits were analyzed via logistic regression. A total of 262 individuals (85.5% females) with a mean age of 36.1 years were included in the analysis. The frequency of migraine attacks was lower in patients in the last DPI tertile compared to those in the first DPI tertile both in the crude [incidence rate ratio (IRR) = 0.70, 95% confidence interval (CI) 0.63, 0.78, P_trend_ < 0.001] and fully-adjusted models (IRR = 0.84, 95% CI 0.74, 0.96, P_trend_ = 0.009). After controlling for potential confounders, an inverse relationship was observed between higher adherence to DPI and migraine-related disability (β = − 2.48, 95% CI − 4.86, − 0.10, P trend = 0.046). After controlling for potential confounders, no significant relationship was observed between DPI and depression (OR = 0.79, 95% CI 0.42, 1.47, P_trend_ = 0.480), anxiety (OR = 1.14, 95% CI 0.61, 2.14, P_trend_ = 0.655), and stress (OR = 1.04, 95% CI 0.57, 1.90, P_trend_ = 0.876). Higher intakes of phytochemical-rich foods may be associated with lower migraine frequency and improved daily activities among patients. Further studies should confirm our observations and delineate the biological pathways linking phytochemicals and migraine headaches.

## Introduction

Migraine is a recurrent neurovascular disorder defined by episodic and unilateral pulsatile headache accompanied by nausea, vomiting, phonophobia, and/or photophobia, and disturbance in quality of life, usual patient activity, and psychological status^1,2^. This leads to a significant economic burden for both individuals and healthcare systems^3^. The prevalence of migraine in the general population has been estimated to be 2.89% in 2008 and 14.0% in 2022^4^. The exact pathophysiology of migraine has not been fully discovered. In addition, current medication therapies for migraine management have limited effectiveness and often come with adverse effects^5–7^. Therefore, it is crucial to identify the determinants of a migraine headache to develop effective management strategies based on its pathophysiology.

Besides genetic predisposition, sex, and age, modifiable risk factors such as diet, obesity, mental health, and physical activity are known to contribute to migraine^8,9^. Namely, dietary behaviors are crucial in migraine management. Previous epidemiological studies have demonstrated the link between healthy eating patterns rich in fruits, vegetables, whole grains, nuts, and olive oil, including the Dietary Approaches to Stop Hypertension (DASH) and Mediterranean eating plans, and migraine-related clinical outcomes^10–16^. Plant-based foods are rich sources of phytochemicals, and their health-related efficacy has been proposed^17,18^. Only a few studies have examined the relationship between dietary phytochemicals and migraine headaches^19,20^, despite the fact that there is biological evidence to suggest a relationship^21–23^. However, these previous studies were limited in scope and size, and have produced contradictory findings. Therefore, it is still unclear whether there is a definitive link between phytochemicals and migraines.

Hence, we leveraged the dietary phytochemical index (DPI), a validated tool, to enhance our understanding of the relationship between phytochemicals and migraine^24^. The DPI represents the percentage of energy intake derived from phytochemical-rich foods, reflecting the total content of phytochemicals in the diet. The primary purpose of our study was to detect a relationship between DPI and clinical outcomes of migraine headaches in a sample of Iranian patients. We evaluated the link between DPI and mental health including depression, anxiety, and stress as our secondary aim. We hypothesized that higher consumption of phytochemical-rich foods could be related with favorable outcomes in patients with migraine.

## Methods

### Study design and participants

This cross-sectional study was conducted between August 2019 and June 2020 at two neurology clinics affiliated with Isfahan University of Medical Sciences, Isfahan, Iran. Together, 265 adults aged 20–50 years with a clinical diagnosis of migraine were included. A board-certified neurologist (F.K) confirmed migraine diagnosis using the International Classification of Headache Disorders-3 (ICHD-3) criteria^25^. A convenience sampling method was used to recruit patients. Convenience sampling is a non-probability sampling method that involves selecting participants based on their easy availability or accessibility to the researcher^26^. In this regard, we selected our participants according to predefined inclusion and exclusion criteria from those who were admitted to both neurology clinics.

Patients were excluded if they (1) had a history of conditions overlapping or interfering with migraine complications, including cardiovascular disease, hypertension, diabetes, cancer, hepatic, renal, thyroid, and other neurological disorders; (2) had a body mass index (BMI) of < 18.5 or ≥ 30 kg/m^2^^27^; or, (3) reported daily energy intakes < 800 kcal/d or > 4200 kcal/d. The risk of migraine is strongly associated with body composition^28,29^; therefore, only normal-weight and overweight participants were enrolled to increase the internal validity of our work. Moreover, the history of the disease was assessed through a face-to-face interview with a physician and an assessment of the patient’s medical documents.

The study protocol was approved by the research ethics committee of Isfahan University of Medical Sciences (IR.MUI.RESEARCH.REC.1398.352). All procedures were conducted in compliance with the Declaration of Helsinki. All patients provided written informed consent before enrollment.

### Dietary assessment

A 168-item semi-quantitative food frequency questionnaire (FFQ) was used to evaluate the usual dietary intake of patients in the preceding year through face-to-face interviews with a registered dietitian. The reliability and validity of FFQ for the Iranian population have been established previously^30–32^. Patients were instructed to report their daily, weekly, and monthly food intake. All portion sizes were converted into grams using household measures^33^. Daily intake of energy and nutrients was subsequently calculated using the Nutritionist IV software (First Databank, Hearst Corp, San Bruno, CA, USA).

### DPI calculations

The DPI was calculated based on the equation suggested by McCarty et al.^24^ as follows: [DPI = energy intake from phytochemical-rich foods (kJ/d)/total energy intake (kJ/day) × 100]. Legumes, nuts, seeds, fruits and vegetables, fruits and vegetable juices, tomato sauces, whole grains, olive, olive oil, and soy sources were included in the calculation. Due to the limited content of phytochemicals in potatoes, we did not consider this item in the DPI calculation.

### Assessments of migraine clinical features

All patients were instructed to complete a 30-day diary to record their migraine clinical traits, including the frequency, duration, and severity of headaches in the upcoming month. Migraine severity was evaluated using the visual analog scale (VAS), wherein the severity was ranked between zero and 10, with “0” indicating no pain and “10” the worst imaginable pain. The validity and reliability of this questionnaire have been approved previously^34^. Further, the Headache Impact Test (HIT-6) was used to evaluate the impact of migraine on the patients’ usual daily activities. The HIT-6 represented a self-administered questionnaire containing six questions. Each question included five response items (never, rarely, sometimes, very often, always), with assigned scores of six, eight, 10, 11, and 13, respectively. The total HIT-6 scores ranged between 36 and 78. The score ranges 36–49, 50–55, 56–59, and ≥ 60 reflected that migraine had minimal, moderate, substantial, or severe impact on patients’ daily activities^35^. It has been confirmed that this questionnaire is valid and reliable among the Iranian population^36^.

### Assessment of psychological characteristics

The Depression, Anxiety, and Stress Scale (DASS-21), a 21-item questionnaire, was used to assess psychological traits. The validity and reliability of the scale among the Iranian population have been previously approved^37^. This questionnaire consisted of seven questions for each subscale of Depression, Anxiety, and Stress. Responses to each question were scored between zero (never) and three (most of the time). The total scores calculated for each subscale were multiplied by two to interpret scores on the original DASS-42 questionnaire. The total score for each subscale of DASS-21 ranged between zero and 42, with higher scores representing higher psychological distress^38^.

### Assessment of other variables

General information including age, sex, marital status, tobacco smoking, family size, migraine type (episodic or chronic), migraine features (with or without aura), family history of migraine diagnosis, time since migraine diagnosis, and medication were collected through face-to-face interviews. To evaluate the physical activity status of patients, a validated International Physical Activity Questionnaire (IPAQ) designed for the Iranian population was used^39^. The questionnaire consisted of seven questions that distinguished the frequency and duration of mild, moderate, and intensive physical activity, as well as inactivity, over seven days. The overall score was reported as MET.h/d. Blood pressure was measured in the sitting position twice using a mercury sphygmomanometer (Riester, Germany) after 10 min of rest, and the average of two measurements was used for further analysis. A digital scale [Omron BF511 (Omron Corp, Kyoto, Japan)] was used for evaluating the patient’s body weight to the nearest 100 g with minimal clothing and no shoes. Height was measured using upstretched tape to the nearest 1 cm in a standing position with relaxed shoulders and no shoes. BMI was calculated using a formula: weight (kg)/height^2^ (m^2^).

A 5 mL fasting blood sample was collected for all patients. Specimens were immediately centrifuged (Avanti J-25, Beckman, Brea, CA, USA) at 3500 rpm for 10 min, and serum was stored at − 80 °C before biochemical analyses. Nitric oxide (NO) levels were assayed using the Griess method and available commercial kits (Kiazist Life Sciences, Iran).

### Statistical analysis

The study sample size was calculated based on the suggested formula for a cross-sectional design consistent with previous studies with α = 0.05, β = 0.95, and r = 0.25. After accounting for an estimated 10% of the drop-out rates, a sample size of n = 265 was deemed adequate^40^. Before data analysis, the normal distribution of continuous variables was examined using Q-Q plots, histogram charts, and skewness statistics. For the skewness statistic, values outside the range of − 2 to + 2 are considered a departure from normality. Since the missing data percentage was small, we restricted analyses to individuals with complete data on all variables. Quantitative and qualitative variables were expressed as mean ± standard error (SE) and frequency (percentage). The difference of continuous variables across DPI tertiles was evaluated by one-way analysis of variance (ANOVA) and Tukey test for post hoc analysis, while the Chi-squared or Fisher’s exact test was utilized for proportions. The relationship between DPI and headache frequency was evaluated using the Poisson regression analysis and an incidence rate ratio (IRR) with a 95% confidence interval (CI) was reported while the other clinical and biochemical outcomes including headache duration, severity, HIT-6, and serum levels of NO were analyzed using linear regression analysis and beta (β) and 95% CI was presented. In addition, the link between psychological traits (depression, anxiety, stress) and DPI was explored using logistic regression analysis, and odds ratio (OR) with 95% CI was reported. All analyses were done in the crude and adjusted models. The first adjusted model accounted for age (continuous), sex (male, female), and total energy intake (continuous). The next model was additionally adjusted for marital status (single, married), current smoking status (yes, no), migraine type (with or without aura), family history (yes, no), mean arterial pressure (continuous), medication use (yes, no), and physical activity (continuous). The last model was additionally adjusted for BMI (continuous). Data were analyzed using SPSS version 21 (IBM Corp, Armonk, NY, USA). All tests were two-tailed and statistically significant at alpha = 0.050.

## Results

### Participants

**Figure 1:**
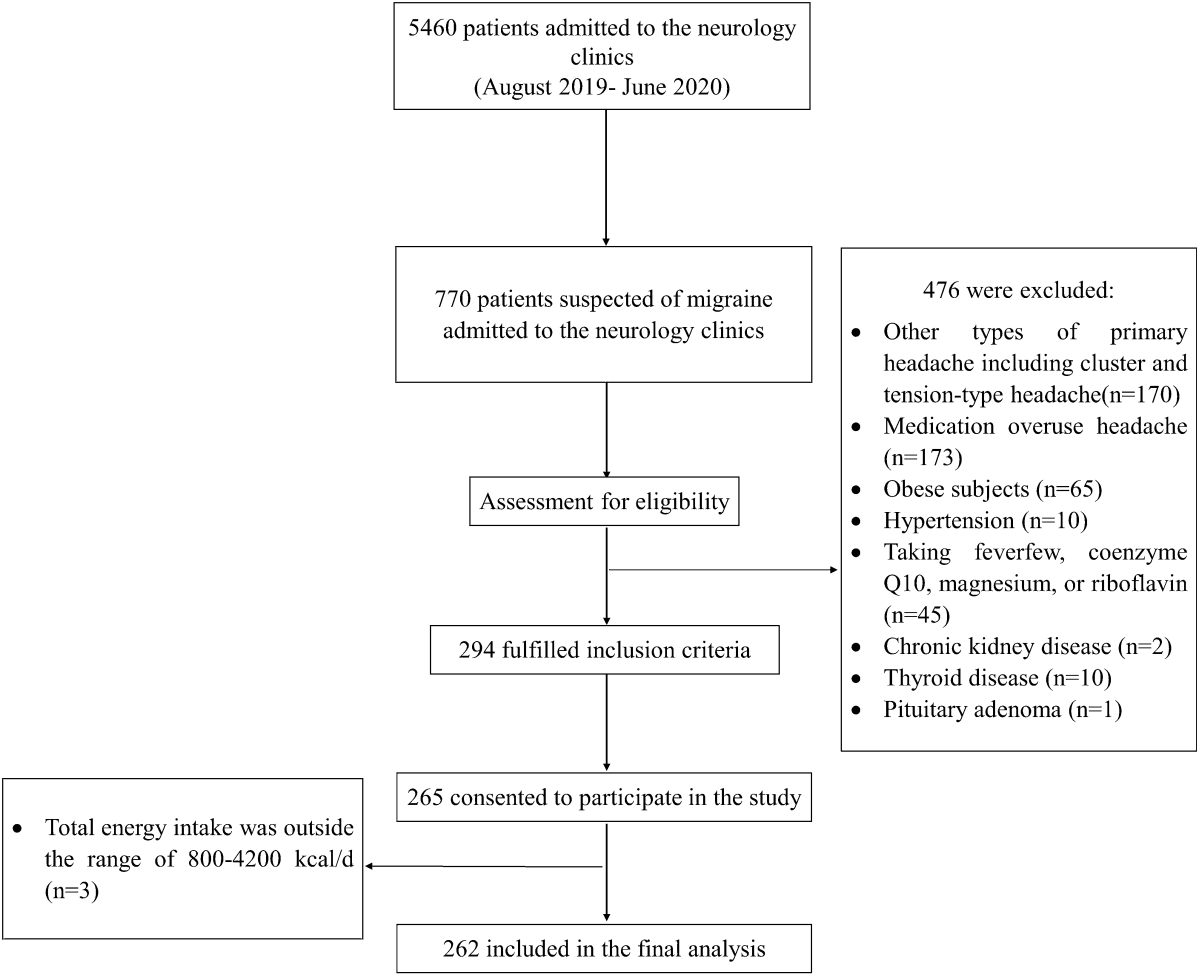
Flow chart of the participants selection process.

Between August 2019 and June 2020, a total of 770 participants were admitted to neurology clinics, of which 476 were excluded. Of the remaining 294 participants, 265 consented to participate in the current study of which an additional three subjects were excluded because their total energy intake, based on FFQ, was outside the range of 800 to 4200 kcal/day (Fig. 1). Overall, 262 patients with migraine, including 224 females and 38 males, were included in the present study. The mean age of the patients was 36.1 (SE: 0.5) years, and the mean BMI was 25.55 (SE: 0.21) kg/m^2^.

### Descriptive data

#### General characteristics

The baseline characteristics of migraine patients across DPI tertiles are summarized in Table 1. The mean age of patients in the third DPI tertile was substantially higher than in the first tertile (*P* = 0.008). Additionally, patients in the highest DPI tertile were more likely females (*P* = 0.010), current smokers (*P* = 0.023), reported migraine headaches with aura (*P* = 0.001), and used benzodiazepines (*P* = 0.037). No significant differences were evident across DPI tertiles for other evaluated measures (all *P* > 0.05; Table 1).

**Table 1:** Characteristics of the study population stratified by tertiles (T) of the dietary phytochemical index (DPI).

Variables	Tertiles of DPI			
	T1 [< 17.99] (n = 87)	T2 [17.99 to 25.93] (n = 88)	T3 [> 25.93] (n = 87)	*P* value
DPI	14.02 ± 0.33	21.93 ± 0.24	32.65 ± 0.58	< 0.001^b^
Demographic variables				
Age (y)	34.9 ± 1.02	35.0 ± 0.83	38.4 ± 0.85	0.008^a^
Sex				0.010
Female	66 (75.9)	80 (90.9)	78 (89.7)	
Male	21 (24.1)	8 (9.1)	10 (10.3)	
Marital status				0.054
Married	66 (75.9)	70 (79.5)	76 (87.4)	
Single	21 (24.1)	18 (20.5)	11 (12.6)	
Current smoker				0.023
Yes	8 (9.2)	6 (6.8)	1 (1.1)	
No	79 (90.8)	82 (93.2)	86 (98.9)	
Number of family members				0.848
1	1 (1.1)	1 (1.1)	0 (0.0)	
2	14 (16.1)	20 (22.7)	15 (17.2)	
3	28 (32.2)	26 (29.5)	31 (35.6)	
+ 4	44 (50.6)	41 (46.6)	41 (47.1)	
Weight (kg)	67.22 ± 1.29	67.56 ± 1.12	68.67 ± 1.01	0.650
Height (cm)	163.83 ± 0.84	162.25 ± 0.83	162.48 ± 0.82	0.350
BMI (kg/m^2^)	24.96 ± 0.37	25.66 ± 0.37	26.02 ± 0.34	0.119
Physical activity (MET.h/d)	9.36 ± 2.86	7.86 ± 1.88	9.42 ± 1.75	0.854
SBP (mmHg)	112.75 ± 0.95	112.04 ± 1.04	113.18 ± 1.11	0.738
DBP (mmHg)	75.66 ± 0.77	74.69 ± 0.83	76.10 ± 0.71	0.422
MAP (mmHg)	88.03 ± 0.75	87.14 ± 0.83	88.46 ± 0.77	0.484
Migraine-related information				
Migraine in first-degree relatives				0.208
Yes	52 (59.8)	55 (62.5)	60 (69.0)	
No	35 (40.2)	33 (37.5)	27 (31.0)	
Time since migraine diagnosis (y)	6.74 ± 0.95	6.29 ± 0.80	8.97 ± 1.00	0.092
Episodic migraine				0.691
Yes	71 (81.6)	72 (81.8)	73 (83.9)	
No	16 (18.4)	16 (18.2)	14 (16.1)	
Migraine with aura				0.001
Yes	26 (29.9)	36 (40.9)	47 (54.0)	
No	61 (70.1)	52 (59.1)	40 (46.0)	
Medications				
Taking beta-blockers				0.539
Yes	36 (41.4)	40 (45.5)	32 (35.8)	
No	51 (58.6)	48 (54.5)	55 (63.2)	
Taking topiramate				0.163
Yes	3 (3.4)	3 (3.4)	7 (8.0)	
No	84 (96.6)	85 (96.6)	80 (92.0)	
Taking TCAs				0.363
Yes	36 (41.4)	44 (50.0)	42 (48.3)	
No	51 (58.6)	44 (50.0)	45 (51.7)	
Taking TeCAs				> 0.99
Yes	4 (4.6)	0 (0.0)	4 (4.6)	
No	83 (95.4)	88 (100.0)	83 (95.4)	
Taking SNRIs				0.313
Yes	6 (6.9)	5 (5.7)	3 (3.4)	
No	81 (93.1)	83 (94.3)	84 (96.6)	
Taking sodium valproate				0.362
Yes	13 (14.9)	11 (12.5)	9 (10.3)	
No	74 (85.1)	77 (87.5)	78 (89.7)	
Taking triptans				0.838
Yes	15 (17.2)	14 (15.9)	14 (16.1)	
No	72 (82.8)	74 (84.1)	73 (83.9)	
Taking gabapentin				0.414
Yes	12 (13.8)	15 (17.0)	16 (18.4)	
No	75 (86.2)	73 (83.0)	71 (81.6)	
Taking benzodiazepine				0.037
Yes	7 (8.0)	5 (5.7)	1 (1.1)	
No	80 (92.0)	83 (94.3)	86 (98.9)	

Data are presented as mean ± standard error or number (% within tertiles of DPI).

*P* value obtained from chi-squared or Fisher’s exact analysis for categorical variables and analysis of variance (ANOVA) for continuous variables.

^a^Significant difference between tertiles 1 and 2 by Tukey post-hoc analysis (*P* < 0.050).

^b^Significant difference between tertiles 1 and 3 by Tukey post-hoc analysis (*P* < 0.050).

^c^Significant difference between tertiles 2 and 3 by Tukey post-hoc analysis (*P* < 0.050).

BMI: Body mass index, SBP: Systolic Blood Pressure; DBP: Diastolic Blood Pressure; MAP: Mean Arterial Pressure; TCA: Tricyclic Antidepressants; TeCA: Tetracyclic Antidepressant; SNRI: Serotonin-Norepinephrine Reuptake Inhibitor, HIT: Headache Impact Test, DPI: Dietary Phytochemical Index.

#### Dietary intakes

Participants’ dietary intakes, including macronutrients and food groups across DPI tertiles, are shown in Table 2. Patients in the highest DPI tertile had a significantly higher carbohydrate intake (*P* = 0.019) and protein (*P* = 0.031) than those in the lowest DPI tertile. In contrast, fat intake was lower in the third DPI tertile than in the first DPI tertile (*P* = 0.043). Also, higher intakes of vegetables, fruits, whole grains, total fruits (fruits plus fruit juices), nuts and legumes, and seafood or plant proteins were observed in patients in the last DPI vs. the first tertile (*P* < 0.001). Nevertheless, lower intakes of refined grains and added sugar were evident in patients in the third DPI vs. the first tertile (*P* < 0.001). Total dairy (*P* = 0.648) and red and processed meat (*P* = 0.845) intakes were comparable across the tertiles (Table 2).

**Table 2:** Selected food groups and nutrient intake of participants across tertiles (T) of the dietary phytochemical index (DPI).

Variables	Tertiles of DPI			
	T1 [< 17.99] (n = 87)	T2 [17.99 to 25.93] (n = 88)	T3 [> 25.93] (n = 87)	*P* value
Energy (Kcal/day)*	2691.96 ± 70.72	2539.57 ± 65.08	2719.16 ± 77.83	0.169
Protein (g/d)	69.68 ± 2.20	73.16 ± 1.45	77.08 ± 2.17	0.031^b^
Fat (g/d)	114.19 ± 2.43	111.87 ± 2.36	106.46 ± 1.81	0.043^b^
Carbohydrate (g/d)	352.53 ± 5.36	359.69 ± 4.94	372.47 ± 4.80	0.019^b^
Food groups (g/day)				
Total vegetables	284.06 ± 14.76	360.99 ± 19.76	449.16 ± 26.04	< 0.001^ab^
Total fruit	338.29 ± 17.27	524.66 ± 17.03	756.53 ± 27.54	< 0.001^ab^
Whole fruit	327.44 ± 16.96	513.83 ± 17.13	738.25 ± 27.94	< 0.001^ab^
Whole grains	52.97 ± 2.41	66.05 ± 3.27	70.49 ± 3.39	< 0.001^ab^
Refined grains	436.67 ± 19.26	397.30 ± 17.91	303.71 ± 13.95	< 0.001^b^
Total dairy	387.66 ± 32.29	425.86 ± 40.21	432.59 ± 39.12	0.648
Nuts and legumes	38.35 ± 3.41	63.86 ± 5.82	60.56 ± 5.22	< 0.001^ab^
Red/processed meat	53.59 ± 5.77	51.71 ± 4.48	56.03 ± 5.21	0.845
Seafood or plant protein	34.10 ± 3.08	54.68 ± 4.61	52.97 ± 4.44	< 0.001^ab^
Added sugars	55.88 ± 5.57	43.13 ± 4.37	28.88 ± 3.41	< 0.001^b^

Data are presented as mean ± standard error and obtained from analysis of variance (ANOVA).

*P* < 0.05 was considered statistically significant.

*Selected food groups and nutrients were adjusted for total energy intake.

^a^Significant difference between tertiles 1 and 2 by Tukey post-hoc analysis (*P* < 0.050).

^b^Significant difference between tertiles 1 and 3 by Tukey post-hoc analysis (*P* < 0.050).

^c^Significant difference between tertiles 2 and 3 by Tukey post-hoc analysis (*P* < 0.050).

DPI: Dietary Phytochemical Index.

#### Clinical characteristics

The mean HIT-6 score (*P* = 0.029) and frequency of migraine attacks (*P* = 0.038) were significantly lower in patients in the highest DPI tertile compared to patients in the lowest tertile. However, no other significant differences were observed in terms of psychological traits and headache duration, severity, and serum levels of NO (Table 3).

**Table 3:** Mental health (depression, anxiety, and stress) and clinical outcomes of migraine headaches across tertiles (T) of dietary phytochemical index (DPI).

	Tertiles of DPI			
	T1 [< 17.99] (n = 87)	T2 [17.99 to 25.93] (n = 88)	T3 [> 25.93] (n = 87)	*P* value
Frequency (attacks per month)	8.91 ± 0.86	8.19 ± 0.82	6.29 ± 0.48	0.038^b^
Duration (day/attack)	0.93 ± 0.08	0.94 ± 0.08	1.02 ± 0.09	0.702
Severity (visual analog scale)	7.54 ± 0.18	7.81 ± 0.19	7.97 ± 0.18	0.263
HIT-6	63.16 ± 0.69	63.88 ± 0.76	61.10 ± 0.81	0.029
Nitric oxide (nmol/mL)	34.99 ± 2.24	32.31 ± 2.36	35.10 ± 2.23	0.618
Depression				0.608
Normal	26 (29.9)	25 (28.4)	32 (36.8)	
Mild	17 (19.5)	14 (15.9)	8 (9.2)	
Moderate	19 (21.8)	23 (26.1)	24 (27.6)	
Severe	6 (6.9)	9 (10.2)	9 (10.3)	
Extremely severe	19 (21.8)	17 (19.3)	14 (16.1)	
Anxiety				0.780
Normal	23 (26.4)	20 (22.7)	26 (29.9)	
Mild	9 (10.3)	4 (4.5)	8 (9.2)	
Moderate	18 (20.7)	18 (20.5)	7 (8.0)	
Severe	7 (8.0)	13 (14.8)	14 (16.1)	
Extremely severe	30 (34.5)	33 (37.5)	32 (36.8)	
Stress				> 0.99
Normal	15 (17.2)	16 (18.2)	22 (25.3)	
Mild	19 (21.8)	13 (14.8)	13 (14.9)	
Moderate	16 (18.4)	16 (18.2)	12 (13.8)	
Severe	21 (24.1)	28 (31.8)	19 (21.8)	
Extremely severe	16 (18.4)	15 (17.0)	21 (24.1)	

Data are presented as mean ± standard error or number (% within tertiles of DPI).

*P* value obtained from chi-squared or Fisher’s exact analysis for categorical variables and analysis of variance (ANOVA) for continuous variables.

^a^Significant difference between tertiles 1 and 2 by Tukey post-hoc analysis (*P* < 0.050).

^b^Significant difference between tertiles 1 and 3 by Tukey post-hoc analysis (*P* < 0.050).

^c^Significant difference between tertiles 2 and 3 by Tukey post-hoc analysis (*P* < 0.050).

HIT: Headache Impact Test, DPI: Dietary Phytochemical Index.

### Main results

#### Clinical outcomes of migraine

The frequency of migraine attacks was lower in patients in the last DPI tertile compared to those in the first DPI tertile both in the crude (IRR = 0.70, 95% CI 0.63, 0.78, P_trend_ < 0.001) and fully-adjusted models (IRR = 0.84, 95% CI 0.74, 0.96, P_trend_ = 0.009). A non-significant relationship was found between DPI and HIT-6 in the crude model (β = − 2.05, 95% CI − 4.15, 0.04, P_trend_ = 0.056). However, the relationship became significant after adjustment for confounders (β = − 2.48, 95% CI − 4.86, − 0.10, P_trend_ = 0.046). No significant relationship was found between DPI and NO, migraine headache duration, and severity in the crude or adjusted models (Table 4).

**Table 4:** Clinical features of migraine headaches according to tertiles (T) of the dietary phytochemical index (DPI).

	Tertiles of DPI		
	T2	T3	*p-trend*
Frequency^1^			
Crude	0.91 (0.83, 1.01)	0.70 (0.63, 0.78)	< 0.001
Model 1	0.91 (0.81, 1.03)	0.74 (0.65, 0.84)	< 0.001
Model 2	0.91 (0.81, 1.03)	0.84 (0.74, 0.96)	0.010
Model 3	0.91 (0.81, 1.02)	0.84 (0.74, 0.96)	0.009
Duration^2^			
Crude	0.01 (− 0.23, 0.25)	0.09 (− 0.14, 0.34)	0.439
Model 1	− 0.04 (− 0.32, 0.22)	0.05 (− 0.22, 0.33)	0.717
Model 2	− 0.009 (− 0.27, 0.25)	0.11 (− 0.16, 0.38)	0.459
Model 3	0.0003 (− 0.26, 0.26)	0.11 (− 0.16, 0.39)	0.439
Severity^2^			
Crude	0.27 (− 0.24, 0.80)	0.43 (− 0.08, 0.96)	0.104
Model 1	0.07 (− 0.47, 0.63)	0.37 (− 0.18, 0.93)	0.199
Model 2	0.06 (− 0.48, 0.61)	0.35 (− 0.21, 0.92)	0.232
Model 3	0.05 (− 0.49, 0.60)	0.35 (− 0.22, 0.92)	0.239
HIT-6^2^			
Crude	0.72 (− 1.36, 2.81)	− 2.05 (− 4.15, 0.04)	0.056
Model 1	− 0.34 (− 2.63, 1.94)	− 2.32 (− 4.65, 0.006)	0.055
Model 2	− 0.43 (− 2.72, 1.84)	− 2.56 (− 4.95, − 0.17)	0.040
Model 3	− 0.25 (− 2.53, 2.02)	− 2.48 (− 4.86, − 0.10)	0.046
Nitric oxide^2^			
Crude	− 2.68 (− 8.97, 3.60)	0.10 (− 6.19, 6.41)	0.973
Model 1	− 1.13 (− 8.10, 5.84)	0.48 (− 6.61, 7.58)	0.908
Model 2	− 1.27 (− 8.21, 5.65)	− 0.70 (− 7.95, 6.55)	0.835
Model 3	− 1.04 (− 8.00, 5.90)	− 0.60 (− 7.85, 6.64)	0.860

Data are presented as β (95% confidence interval) and obtained from linear regression^2^ or incidence-rate ratio (95% confidence interval) obtained from Poisson regression^1^.

Tertile 1 was considered as a reference.

*P* < 0.05 was considered statistically significant.

Crude: Unadjusted.

Model 1: Adjusted for age, sex, and energy intake.

Model 2: Model 1 + marital status, smoking status, migraine type, family history, mean arterial pressure, medication, and physical activity.

Model 3: Model 2 + body mass index.

HIT: Headache Impact Test, DPI: Dietary Phytochemical Index.

#### Physiological traits

Crude and multivariate-adjusted OR and 95% CIs for depression, anxiety, and stress are shown in Table 5. After controlling for potential confounders, no significant relationship was observed between DPI and depression (OR = 0.79, 95% CI 0.42, 1.47, P_trend_ = 0.480), anxiety (OR = 1.14, 95% CI 0.61, 2.14, P_trend_ = 0.655), and stress (OR = 1.04, 95% CI 0.57, 1.90, P_trend_ = 0.876).

**Table 5:** Mental health (depression, anxiety and stress) of the study population according to tertiles (T) of dietary phytochemical index (DPI).

	Tertiles of dietary phytochemical index		
	T2	T3	*p-trend*
Depression			
Crude	1.07 (0.63, 1.81)	0.87 (0.51, 1.48)	0.616
Model 1	1.01 (0.56, 1.81)	0.82 (0.44, 1.53)	0.558
Model 2	1.01 (0.56, 1.81)	0.79 (0.42, 1.48)	0.497
Model 3	1.004 (0.55, 1.80)	0.79 (0.42, 1.47)	0.480
Anxiety			
Crude	1.28 (0.75, 2.18)	1.07 (0.63, 1.84)	0.772
Model 1	1.19 (0.66, 2.13)	1.11 (0.60, 2.05)	0.712
Model 2	1.17 (0.65, 2.11)	1.15 (0.61, 2.15)	0.637
Model 3	1.15 (0.63, 2.08)	1.14 (0.61, 2.14)	0.655
Stress			
Crude	1.12 (0.67, 1.87)	1.01 (0.59, 1.73)	0.933
Model 1	1.06 (0.60, 1.88)	1.04 (0.57, 1.88)	0.877
Model 2	1.02 (0.58, 1.82)	1.04 (0.57, 1.90)	0.879
Model 3	1.03 (0.58, 1.83)	1.04 (0.57, 1.90)	0.876

Data are presented as odds ratio (95% confidence interval) and obtained from logistic regression.

*P* < 0.05 was considered statistically significant.

Tertile 1 was considered as reference.

Crude: Unadjusted.

Model 1: Adjusted for age, sex, and energy intake.

Model 2: Model 1 + marital status, smoking status, number of family members, mean arterial pressure, medication, and physical activity.

Model 3: Model 2 + body mass index.

## Discussion

In this study, we aimed to evaluate the relationship between DPI and clinical, psychological, and biochemical traits of migraine. The most significant finding of the present work was that patients with higher intakes of phytochemical-rich foods presented with lower migraine frequency and improved migraine-related disability, as assessed by the HIT-6. Nevertheless, we observed no relationship between DPI and other clinical outcomes (duration and severity of headaches), psychological characteristics, and serum NO levels. It is important to note the possibility of bidirectionality when interpreting our findings. Migraine sufferers with higher headache frequencies may omit more food triggers due to nausea and vomiting accompanying their migraines. This could result in lower DPI scores for these patients. Therefore, future longitudinal and prospective studies are necessary to investigate this correlation.

Previous studies have demonstrated an inverse relationship between dietary patterns rich in phytochemicals, such as Mediterranean and DASH diet, and some migraine symptoms, such as severity, frequency, and duration of migraine attacks^41,42^. Moreover, two recent cross-sectional studies have investigated the relationship between DPI and migraine headaches^19,20^, of which results of a survey by Askarpour and co-workers on 66 women aged 18–50 years showed 33% lower odds of headache severity in patients with higher DPI scores. However, no significant relationship was found between DPI and migraine attack duration^19^. Results of the other study on 90 patients with episodic migraines revealed a negative correlation between DPI and the severity of migraine attacks, without relationship between DPI scores and disability as evidenced by the Migraine Disability Assessment Score (MIDAS) or the duration of migraine attacks^20^. Contrary to the results of the studies mentioned above, we observed no relationship between DPI and the severity of migraine headaches. The conflicting results may be partially explained by differences in the studied population, statistical methods used, and covariates considered in the analyses, warranting further investigations to confirm the link and delineate the underlying explanatory mechanism.

Several studies have examined the relationship between DPI and mental health disorders. However, to our knowledge, none have targeted patients with migraine^43–45^. Accordingly, a lower risk of depression, anxiety, and stress has been reported in individuals with the highest vs. lowest DPI scores. We were unable to find studies that investigated the relationship between DPI and psychiatric outcomes in patients with migraine, highlighting a persistent research gap in this critical area. The null findings in our work may be explained, at least in part, by the marginal effect estimates because our study was designed to examine psychological indices as a secondary outcome. Future studies are required to evaluate the link between dietary intake of phytochemicals and psychological trials in patients with migraine.

Several potential mechanisms may underlie the relationship between phytochemicals and migraine headaches. Recent evidence from animal models and human studies suggests that gut microbiota dysbiosis contributes to the pathophysiology of migraine headaches^46^. Therefore, the therapeutic effects of improved gut microbiota, secondary to dietary modifications, on migraine outcomes have biological plausibility. Some reports indicate that there is a favorable link between dietary intakes of phytochemical-rich foods and gut microbiota. Subsequently, these natural bioactive compounds potentially alleviate migraine symptoms by maintaining gut microbiota homeostasis. However, the underlying molecular mechanisms are not well understood^23^. Furthermore, an increased vulnerability to oxidative stress, which results from the synthesis of oxidants, has been implicated in patients with migraine^47,48^. Plant-based foods’ antioxidant properties are mainly due to phytochemicals such as beta-carotene and polyphenols^49^. Phytochemicals may improve migraine symptoms by scavenging excess reactive oxygen (ROS) and nitrogen (RNS) spices and reducing the risk of oxidative stress^22^.

This study has certain limitations that require consideration. Due to the cross-sectional nature of the study, it was not feasible to establish a causal relationship between DPI and the outcomes under consideration. As a result, future large-scale prospective studies are necessary to investigate the causal link between the intake of foods high in phytochemicals and the clinical outcomes of migraine. In addition, because the DPI was calculated based on the energy provided by phytochemical-rich foods and not all phytochemical-rich foods provide energy, certain foods with high phytochemical content, such as tea and coffee, were excluded from the study. Despite using a validated FFQ to evaluate dietary intake, recall bias may still influence the findings. Finally, the study’s small sample size limits its generalizability to patients in other regions of Iran or other countries.

In conclusion, we found that adherence to a phytochemical-rich diet could be associated with favorable clinical outcomes for migraine patients including headache frequency and migraine-related disability. Owing to the cross-sectional nature of the current study, we cannot provide any practical implications regarding phytochemicals and migraine. These findings can be used by other researchers to conduct further well-designed longitudinal studies to confirm our findings and also elaborate our understanding regarding the causality between phytochemicals and migraine. Moreover, further studies are also needed to investigate underlying mechanisms regarding phytochemicals and migraine.

## Data Availability

The data that support the findings of this study are available from the corresponding author upon reasonable request.

## Abbreviations

DPI: Dietary phytochemical index
NO: Nitric oxide
SQ-FFQ: Semi-quantitative food frequency questionnaire
HIT-6: Headache impact test-6
CI: Confidence interval
ICHD-3: The third edition of International Classification for Headache Disorders
BMI: Body mass index
STROBE: Strengthening the Reporting of Observational Studies in Epidemiology
VAS: Visual analog scale
PA: Physical activity
IPAQ: International Physical Activity Questionnaire
ANOVA: Analysis of variance
DASS: Depression, Anxiety, and Stress Scale
